# One-year outcome of a prospective trial stopping dual antiplatelet therapy at 3 months after everolimus-eluting cobalt-chromium stent implantation: ShortT and OPtimal duration of Dual AntiPlatelet Therapy after everolimus-eluting cobalt-chromium stent (STOPDAPT) trial

**DOI:** 10.1007/s12928-015-0366-9

**Published:** 2015-10-30

**Authors:** Masahiro Natsuaki, Takeshi Morimoto, Erika Yamamoto, Hiroki Shiomi, Yutaka Furukawa, Mitsuru Abe, Koichi Nakao, Tetsuya Ishikawa, Kazuya Kawai, Kei Yunoki, Shogo Shimizu, Masaharu Akao, Shinji Miki, Masashi Yamamoto, Hisayuki Okada, Kozo Hoshino, Kazushige Kadota, Yoshihiro Morino, Keiichi Igarashi, Kengo Tanabe, Ken Kozuma, Takeshi Kimura

**Affiliations:** Division of Cardiology, Saiseikai Fukuoka General Hospital, Fukuoka, Japan; Department of Clinical Epidemiology, Hyogo College of Medicine, Nishinomiya, Japan; Department of Cardiovascular Medicine, Graduate School of Medicine, Kyoto University, 54 Shogoin Kawahara-cho, Sakyo-ku, Kyoto, 606-8507 Japan; Department of Cardiovascular Medicine, Kobe City Medical Center General Hospital, Kobe, Japan; Division of Cardiology, National Hospital Organization Kyoto Medical Center, Kyoto, Japan; Division of Cardiology, Saiseikai Kumamoto Hospital, Kumamoto, Japan; Division of Cardiology, Saitama Cardiovascular and Respiratory Center, Kumagaya, Japan; Division of Cardiology, Chikamori Hospital, Kochi, Japan; Division of Cardiology, Osaka City General Hospital, Osaka, Japan; Division of Cardiology, Mashiko Hospital, Kawaguchi, Japan; Division of Cardiology, Mitsubishi Kyoto Hospital, Kyoto, Japan; Division of Cardiology, Kimitsu Chuo Hospital, Kimitsu, Japan; Division of Cardiology, Seirei Hamamatsu General Hospital, Hamamatsu, Japan; Division of Cardiology, Nagai Hospital, Tsu, Japan; Department of Cardiology, Kurashiki Central Hospital, Kurashiki, Japan; Division of Cardiology, Iwate Medical University Hospital, Morioka, Japan; Division of Cardiology, Hokkaido Social Insurance Hospital, Sapporo, Japan; Division of Cardiology, Mitsui Memorial Hospital, Tokyo, Japan; Division of Cardiology, Teikyo University Hospital, Tokyo, Japan

**Keywords:** Dual antiplatelet therapy, Everolimus-eluting stent, Coronary artery disease, Coronary stent

## Abstract

There has been no previous prospective study evaluating dual antiplatelet therapy (DAPT) duration shorter than 6 months after cobalt-chromium everolimus-eluting stent (CoCr-EES) implantation. STOPDAPT trial is a prospective multi-center single-arm study evaluating 3-month DAPT duration after CoCr-EES implantation. The primary endpoint was a composite of cardiovascular death, myocardial infarction (MI), stroke, definite stent thrombosis (ST) and TIMI major/minor bleeding at 1 year. Between September 2012 and October 2013, a total of 1525 patients were enrolled from 58 Japanese centers, with complete 1-year follow-up in 1519 patients (99.6 %). Thienopyridine was discontinued within 4 months in 1444 patients (94.7 %). The event rates beyond 3 months were very low (cardiovascular death: 0.5 %, MI: 0.1 %, ST: 0 %, stroke: 0.7 %, and TIMI major/minor bleeding: 0.8 %). Cumulative 1-year incidence of the primary endpoint was 2.8 % [upper 97.5 % confidence interval (CI) 3.6 %], which was lower than the pre-defined performance goal of 6.6 % (*P* < 0.0001). Using the CoCr-EES group in the RESET trial as a historical comparison group, where nearly 90 % of patients had continued DAPT at 1 year, cumulative incidence of the primary endpoint tended to be lower in the STOPDAPT than in the RESET (2.8 versus 4.0 %, *P* = 0.06) and adjusted hazard ratio was 0.64 (95 % CI 0.42–0.95, *P* = 0.03). The cumulative incidence of definite/probable ST was lower in the STOPDAPT than in the RESET [0 patient (0 %) versus 5 patients (0.3 %), *P* = 0.03]. In conclusion, stopping DAPT at 3 months in selected patients after CoCr-EES implantation was at least as safe as the prolonged DAPT regimen adopted in the historical control group.

**Electronic supplementary material:**

The online version of this article (doi:10.1007/s12928-015-0366-9) contains supplementary material, which is available to authorized users.

## Introduction

Several previous randomized controlled trials comparing short (3–6 months) dual antiplatelet therapy (DAPT) with pro1onged (12 months or longer) DAPT after coronary stent implantation demonstrated similar ischemic event risk and lower bleeding event risk with shorter course of DAPT [1–5]. Therefore, the current ESC/EACTS guideline recommend 6-month DAPT after new generation coronary drug-eluting stent (DES) implantation in patients with stable coronary artery disease [6]. Two previous trials (RESET and OPTIMIZE) suggested the safety and efficacy of 3-month DAPT after implantation of one of the first generation (G1) DES, Endeavor™ zotarolimus-eluting stent (E-ZES), which was associated with relatively large late lumen loss (neointimal hyperplasia) similar to bare-metal stents (BMS) [3, 4]. Second-generation drug-eluting stent (G2-DES) with small late lumen loss, cobalt-chromium everolimus-eluting stent (CoCr-EES) in particular, has been reported to have lower risk for stent thrombosis (ST) compared with G1-DES or BMS [7]. Therefore, the optimal DAPT duration after G2-DES implantation could be shorter than 6–12 months currently recommended in the guidelines [6, 8]. However, there has been no previous prospective study evaluating DAPT duration shorter than 6 months after G2-DES implantation.

In the current study, we sought to evaluate the safety of 3-month DAPT duration after CoCr-EES implantation in a prospective multicenter single-arm trial.

## Methods

### Study population

ShortT and OPtimal duration of Dual AntiPlatelet Therapy after everolimus-eluting cobalt-chromium stent (STOPDAPT) trial is a prospective multi-center single-arm trial enrolling patients who agreed to follow the 3-month DAPT protocol (discontinuation of clopidogrel at 2–4 months and aspirin monotherapy thereafter) after successful CoCr-EES implantation. Patients who underwent successful percutaneous coronary intervention (PCI) using CoCr-EES were to be enrolled, if the physicians in charge judged 3-month DAPT duration to be appropriate for the patient. Patients who had previous history of PCI using DES other than CoCr-EES were excluded. The study sponsor (Abbott vascular) was involved in the discussion on the study design, and gave final approval for submission of the manuscript. However, patient enrollment, data collection, statistical analysis, and manuscript preparation were conducted independent of the study sponsor. The relevant review boards or ethics committees in all participating centers approved the research protocol. The trial was registered with ClinicalTrials.gov number, NCT01303640.

**Fig. 1:**
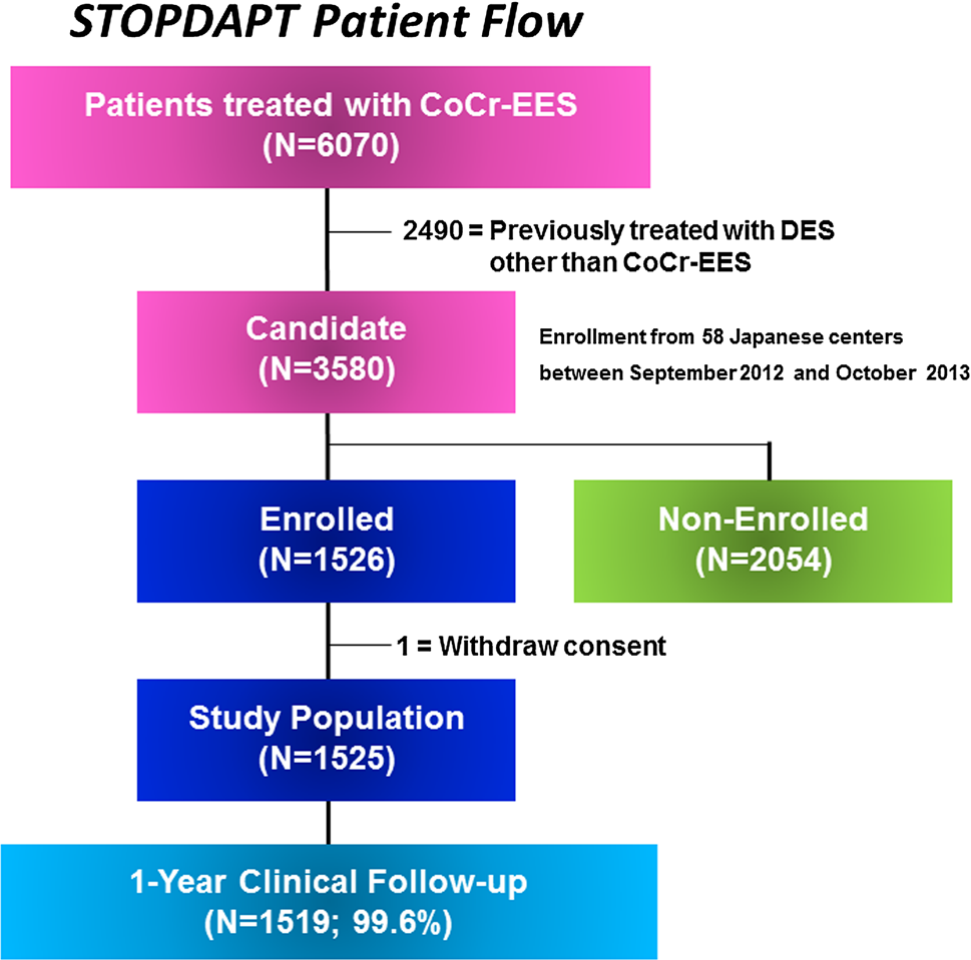
Study flow chart. CoCr-EES, Cobalt-chromium everolimus-eluting stent; DES, drug-eluting stent

Between September 2012 and October 2013, 6070 patients underwent PCI using CoCr-EES in 58 Japanese centers (Supplemental Appendix A). We excluded 2490 patients who were previously treated with DES other than CoCr-EES. Among 3580 eligible patients, 1526 patients (43 %) were enrolled in this study. Excluding 1 patient who withdrew consent for study participation, 1525 patients constituted the current study population (Fig. 1). Among 2054 patients who were not enrolled in this study, 62 % of patients were judged by the attending physicians not suitable for the study and 14 % of patients refused study participation (Table 1).

**Table 1:** Baseline characteristics: enrolled versus non-enrolled patients

	Enrolled (*N* = 1525)	Non-enrolled (*N* = 2054)	*P* value
Age (years)	70.0 ± 10.6	70.0 ± 11.0	0.97
Age ≥75 years	570 (37 %)	776 (38 %)	0.81
Male gender	1117 (73 %)	1553 (76 %)	0.11
Body mass index	24.1 ± 3.6	23.9 ± 3.6 (2010)	0.04
Coexisting condition			
Hypertension	1260 (83 %)	1574 (77 %)	<0.0001
Diabetes mellitus	604 (40 %)	824 (40 %)	0.76
Insulin-treated diabetes	119 (7.8 %)	176 (8.6 %)	0.41
Treated with oral medication only	360 (24 %)	482 (23 %)	0.92
Treated with diet therapy only	125 (8.2 %)	166 (8.1 %)	0.9
ESRD (eGFR < 30 mL/min/1.73 m^2^) not on hemodialysis	35/1521 (2.3 %)	93/2054 (4.5 %)	0.0003
Hemodialysis	56 (3.7 %)	141 (6.9 %)	<0.0001
Cardiac risk factor			
Current smoker	315 (21 %)	430 (21 %)	0.84
Prior Stroke	168 (11 %)	243 (12 %)	0.45
Heart failure	101 (6.6 %)	191 (9.3 %)	0.004
Peripheral vascular disease	142 (9.3 %)	177 (8.6 %)	0.47
Clinical characteristics			
Clinical presentation			
Stable coronary artery disease	1040 (68 %)	1277 (62 %)	0.0002
Unstable angina	229 (15 %)	299 (15 %)	0.7
Acute myocardial infarction	256 (17 %)	478 (23 %)	<0.0001
Target-vessel location			
Left main coronary artery	17 (1.1 %)	160 (7.8 %)	<0.0001
Left anterior descending coronary artery	866 (57 %)	1108 (54 %)	0.09
Left circumflex coronary artery	361 (24 %)	460 (22 %)	0.37
Right coronary artery	405 (27 %)	614 (30 %)	0.03
Bypass graft	4 (0.3 %)	17 (0.8 %)	0.02
Complexity of coronary artery disease			
Number of treated lesions per patient	1.21 ± 0.48	1.43 ± 0.74	<0.0001
Multi-vessel treatment	130 (8.5 %)	315 (15 %)	<0.0001
Reasons for non-enrollment			
Physicians’ judgment not to be suitable for the study	NA	1276 (62 %)	
Patients’ refusal for study participation	NA	292 (14 %)	
Others	NA	486 (24 %)	

Values are expressed as mean ± SD or number (%)

*ESRD* end stage renal disease, *eGFR* estimated glomerular filtration rate

As a historical control group, we selected the CoCr-EES group in the RESET (Randomized Evaluation of Sirolimus-eluting versus Everolimus-eluting stent Trial) trial (a randomized controlled trial comparing CoCr-EES with sirolimus-eluting stent conducted by the same study group in 2010), where nearly 90 % of patients had continued DAPT at 1 year [9]. The eligibility criteria of the RESET were comparable to that of the STOPDAPT except for the inclusion of patients with previous DES implantation. Among 1597 patients in the CoCr-EES group in the RESET, 38 patients with in-hospital primary endpoint events were excluded from the historical control group in this study, because patients in the STOPDAPT were enrolled after completion of successful PCI. A total of 1559 patients were selected as a historical control group.

### Procedures

Antiplatelet regimen included aspirin (≥81 mg daily) indefinitely and thienopyridine (75 mg clopidogrel daily) for 3 months after stent implantation. Ticlopidine 200 mg/day was only allowed for those who did not tolerate clopidogrel. Patients were instructed to discontinue thienopyridine at 3-month hospital visit. Acceptable time window for the discontinuation of thienopyridine therapy was within ±1 month. Status of antiplatelet therapy was evaluated throughout the follow-up period as previously described [10]. Persistent discontinuation of thienopyridine was defined as withdrawal lasting for at least 2 months [10].

### Endpoints and definitions

The primary endpoint in this trial was a composite of cardiovascular death, myocardial infarction (MI), stroke, definite ST and Thrombolysis in Myocardial Infarction (TIMI) major/minor bleeding at 1 year. Primary endpoint events were adjudicated by the independent clinical event committee (Supplemental Appendix B). Major secondary endpoints were TIMI major/minor bleeding and a composite of cardiovascular death, MI, stroke or definite ST at 1 year. Secondary endpoints included death, MI, stroke, possible/probable/definite ST, TIMI/Global Utilization of Streptokinase and Tissue plasminogen activator for Occluded coronary arteries (GUSTO) bleeding, target-lesion revascularization (TLR), target-vessel revascularization (TVR), coronary artery bypass grafting, and any coronary revascularization.

Death was regarded as cardiac in origin unless obvious non-cardiac causes could be identified. MI and ST were defined according to the Academic Research Consortium definitions [11]. Stroke during follow-up was defined as ischemic or hemorrhagic stroke requiring hospitalization with symptoms lasting >24 h. Bleeding was defined according to the TIMI [12] and GUSTO classifications [13]. TLR was defined as either PCI or coronary artery bypass grafting due to restenosis or thrombosis of the target lesion that included the proximal and distal edge segments as well as the ostium of the side branches.

### Data collection and follow-up

Demographic, angiographic, and procedural data were collected from hospital charts or databases in each participating center according to the pre-specified definitions by experienced clinical research coordinators in the participating centers (Supplemental Appendix B) or in the study management center (Supplemental Appendix B). Follow-up data on the clinical events were collected from the hospital charts in the participating centers (74 %), letters to patients (20 %), and telephone call to referring physicians (8.4 %).

### Angiographic analysis

For the STOPDAPT, qualitative and quantitative coronary angiography was evaluated at the same angiographic core laboratory as in the RESET (Cardiocore, Tokyo, Japan) with use of CAAS 5.9 (Pie Medical Imaging, Maastricht, The Netherlands). Baseline angiograms in the STOPDAPT were assessed in 350 patients randomly selected at the time of enrollment. The target segment was defined as the entire segment involving the implanted stent and the 5-mm proximal and distal edges adjacent to the stent. A segment to be treated with multiple overlapping stents was regarded as a single target segment. In addition to the standard angiographic parameters, SYNTAX (Synergy Between Percutaneous Coronary Intervention With Taxus and Cardiac Surgery) score was also evaluated [14].

### Statistical analysis

The event rate for the primary endpoint in this single-arm trial was compared against a pre-specified performance goal using an exact test through the binominal distribution. To determine the sample size in this study, we used the data from the 1559 patients in the CoCr-EES group in the RESET trial [9]. Actual event rate of the CoCr-EES group in the RESET trial was 4 % and its upper one-sided 80 % confidence limit was 4.4 %. We assumed the true rate 4.4 % and we set the performance goal to be 6.6 % by adding delta of 2.2 % (50 % of 4.4 %) to 4.4 % of true rate. A total of 1455 patients would yield 95 % power at a level of one-sided type 1 error of 0.025 to achieve 6.6 % of performance goal. We finally rounded up to 1500 patients to take into account for dropouts.

Categorical variables were presented as number and percentage and continuous variables were expressed as mean value ± SD or median with inter-quartile range. We used the exact binomial test to compare the incidence of primary endpoint to the performance goal of 6.6 % using one-sided alpha of 0.025. Then, we compared the STOPDAPT group with the RESET group using the Chi-square test or Fisher’s exact test for categorical variables, and Student’s *t* test or Wilcoxon rank sum test based on their distributions for continuous variables. Cumulative incidence was estimated by the Kaplan–Meier method and differences were assessed with the log-rank test. To evaluate the events beyond 3 months, we also conducted the landmark analyses at 3 months. Those patients who had the individual endpoint events before 3 months were excluded in the landmark analyses. Due to the presence of differences in baseline characteristics between the 2 studies, we also used Cox proportional hazard models to estimate the risk of the STOPDAPT relative to the RESET for the primary endpoint. In the multivariable analysis, we chose 10 clinically relevant factors indicated in Table 1 as the risk adjusting variables. The continuous variables were dichotomized by clinically meaningful reference values or median values. The study (STOPDAPT or RESET) and the 10 risk adjusting variables were simultaneously included in the Cox proportional hazard model. The effect of the STOPDAPT compared to the RESET was expressed as hazard ratios (HR) and their 95 % confidence intervals (CI). In the pre-specified sub-group analysis, we also conducted the formal interaction test between the study and subgroup factors.

Statistical analyses were conducted by a physician (Natsuaki M) and by a statistician (Morimoto T) with the use of JMP 10.0 and SAS 9.4 (SAS Institute Inc, Cary, NC, USA) software. We used one-sided *P* values <0.025 as statistically significant level in the evaluation of performance goal, and two-sided *P* values <0.05 as statistically significant for other comparisons.

## Results

### Baseline Characteristics: Enrolled versus Non-enrolled Patients in the STOPDAPT

Baseline characteristics were significantly different in several aspects between the enrolled and non-enrolled patients (Table 1). Chronic kidney disease, hemodialysis, heart failure, and acute myocardial infarction (AMI) presentation were more prevalent in the non-enrolled group, while higher body mass index (BMI) and hypertension were more often found in the enrolled group. Patients with treatment of left main coronary artery were less often enrolled in the study. Regarding the complexity of coronary artery disease, the number of treated lesions was greater and multi-vessel treatment was more often performed in the non-enrolled group than in the enrolled group (Table 1).

### Baseline characteristics: STOPDAPT versus RESET

Baseline characteristics were also significantly different in several aspects between the STOPDAPT and RESET (Table 2). Patients in the STOPDAPT were significantly older than those in the RESET. Female gender, hypertension, dyslipidemia, atrial fibrillation, anemia, and AMI presentation were more often found in the STOPDAPT than in the RESET, while diabetes, hemodialysis, family history of coronary artery disease, prior MI, heart failure, prior PCI, and multi-vessel disease were more prevalent in the RESET than in the STOPDAPT. Patients with treatment of left main coronary artery and chronic total occlusion were less often enrolled in the STOPDAPT than in the RESET. Total stent length per patient was significantly longer in the STOPDAPT, while multi-vessel treatment was more often performed in the RESET. Regarding the medications at hospital discharge, β-blockers and anticoagulants were more often prescribed in the STOPDAPT than in the RESET (Table 2).

**Table 2:** Baseline Characteristics: STOPDAPT versus RESET

	STOPDAPT (*N* = 1525)	RESET (*N* = 1559)	*P* value
Age (years)	70.0 ± 10.6	68.9 ± 9.7	0.002
Age ≥75 years^a^	570 (37 %)	480 (31 %)	0.0001
Male gender^a^	1117 (73 %)	1213 (78 %)	0.003
Body mass index	24.1 ± 3.6	24.3 ± 3.6 (1542)	0.25
Coexisting condition			
Hypertension^a^	1261 (83 %)	1238 (79 %)	0.02
Diabetes mellitus^a^	604 (40 %)	707 (45 %)	0.001
Insulin-treated diabetes	119 (7.8 %)	171 (11 %)	0.003
Treated with oral medication only	360 (24 %)	343 (22 %)	0.29
Treated with diet therapy only	125 (8.2 %)	193 (12 %)	0.0001
Dyslipidemia	1209 (79 %)	1164 (75 %)	0.002
ESRD (eGFR < 30 mL/min/1.73 m^2^) not on hemodialysis	35/1521 (2.3 %)	31/1552 (2.0 %)	0.56
Hemodialysis^a^	56 (3.7 %)	90 (5.8 %)	0.006
Atrial fibrillation	172 (11 %)	104 (6.7 %)	<0.0001
Anemia (hemoglobin <11.0 g/dL)^a^	241 (16 %)	190 (12 %)	0.004
Cardiac risk factor			
Current smoker	315 (21 %)	329 (21 %)	0.76
Family history of coronary artery disease	192 (13 %)	248/1343 (18 %)	<0.0001
Prior myocardial infarction	267 (18 %)	459 (29 %)	<0.0001
Prior stroke^a^	168 (11 %)	176 (11 %)	0.81
Heart failure	101 (6.6 %)	138 (8.9 %)	0.02
Peripheral vascular disease	142 (9.3 %)	140 (9.0 %)	0.75
Prior percutaneous coronary intervention	468 (31 %)	741 (48 %)	<0.0001
Prior coronary artery bypass grafting	41 (2.7 %)	61 (3.9 %)	0.06
Clinical characteristics			
Clinical presentation			
Stable coronary artery disease	1040 (68 %)	1282 (82 %)	<0.0001
Unstable angina	229 (15 %)	175 (11 %)	0.002
Acute myocardial infarction^a^	256 (17 %)	102 (6.5 %)	<0.0001
Left ventricular ejection fraction <30 %	17/1315 (1.3 %)	24/1345 (1.8 %)	0.3
Multi-vessel disease	578 (38 %)	759 (49 %)	<0.0001
Target-vessel location			
Left main coronary artery^a^	17 (1.1 %)	46 (3.0 %)	0.0002
Left anterior descending coronary artery	866 (57 %)	762 (49 %)	<0.0001
Left circumflex coronary artery	361 (24 %)	393 (25 %)	0.32
Right coronary artery	405 (27 %)	511 (33 %)	0.0002
Bypass graft	4 (0.3 %)	6 (0.4 %)	0.55
Complexity of coronary artery disease			
Number of treated lesions per patient	1.21 ± 0.48	1.23 ± 0.51	0.16
Medications			
Aspirin	1524 (99.9 %)	1553 (99.6 %)	0.049
Thienopyridines	1522 (99.8 %)	1552 (99.6 %)	0.21
Clopidogrel	1508 (99.1 %)	1350 (87 %)	<0.0001
Ticlopidine	14 (0.9 %)	200 (13 %)	
Statins	1223 (80 %)	1207 (77 %)	0.06
B-blockers	620 (41 %)	566 (36 %)	0.01
ACE-I/ARB	939 (62 %)	967 (62 %)	0.8
Calcium-channel blockers	675 (44 %)	670 (43 %)	0.47
Nitrates	219 (14 %)	426 (27 %)	<0.0001
Anticoagulants^a^	168 (11 %)	125 (8.0 %)	0.005
Warfarin	125 (8.2 %)	125 (8.0 %)	
Dabigatran	34 (2.2 %)	0 (0 %)	
Rivaroxaban	9 (0.6 %)	0 (0 %)	
Lesion and Procedural characteristics			
Before index procedure			
Chronic total occlusion	72 (4.7 %)	109 (7.0 %)	0.007
Culprit for STEMI	203 (13 %)	69 (4.4 %)	<0.0001
Bifurcation	317 (21 %)	337 (22 %)	0.57
After index procedure			
Number of stents used per patient	1.37 ± 0.65	1.5 ± 0.77 (1554)	<0.0001
Total stent length per patient (mm)	32.9 ± 20.9	30.8 ± 18.9 (1554)	0.004
Multi-vessel treatment	130 (8.5 %)	183 (12 %)	<0.0001

Values are expressed as mean ± SD or number (%)

*ESRD* end stage renal disease, *eGFR* estimated glomerular filtration rate, *ACE-I* angiotensin converting enzyme inhibitors, *ARB* angiotensin II receptor blockers, *STEMI* ST-segment elevation myocardial infarction

^a^Potential independent variables selected for multivariable analysis

### Angiographic characteristics: STOPDAPT versus RESET

In angiographic characteristics, thrombus and bifurcation lesions were more often found in the STOPDAPT, while in-stent restenosis was more prevalent in the RESET. Lesion length was significantly longer and reference vessel diameter was significantly larger in the STOPDAPT than in the RESET. There were small, but significant differences in in-segment minimum lumen diameter, in-segment percent diameter stenosis, and in-segment acute gain between the 2 groups. SYNTAX score was not significantly different between the 2 groups (Table 3).

**Table 3:** Baseline angiographic characteristics: STOPDAPT versus RESET

	STOPDAPT (*N* = 350)	RESET (*N* = 1744)	*P* value
Before index procedure			
Lesion length, mm	19.7 ± 12.6 (307)	17.0 ± 11.5 (1643)	0.0001
Reference vessel diameter, mm	2.69 ± 0.56	2.58 ± 0.63 (1737)	0.002
Minimum lumen diameter, mm	0.8 ± 0.44	0.82 ± 0.48	0.6
Percent diameter stenosis, %	70.1 ± 15.1	69.1 ± 16.4 (1743)	0.27
Thrombus	37 (11 %)	78 (4.5 %)	<0.0001
Chronic total occlusion	12/349 (3.4 %)	72/1725 (4.2 %)	0.52
In-stent restenosis	13 (3.7 %)	192 (11 %)	<0.0001
Bifurcation	176 (50 %)	681 (39 %)	0.0001
Moderate or heavy calcification	74 (21 %)	346 (20 %)	0.58
Small vessel (reference vessel diameter ≤2.75 mm)	189/350 (54 %)	1114/1737 (64 %)	0.0004
Long lesion (lesion length >18 mm)	124/307 (40 %)	559/1643 (34 %)	0.03
SYNTAX score	9 (6–15) (346)	10 (6–16) (1458)	0.06
After index procedure			
Number of stents used			
Per lesion	1.16 ± 0.41 (350)	1.27 ± 0.57 (1743)	0.0008
Bifurcation 2-stent approach	6 (1.7 %)	18 (1.0 %)	0.3
Minimum lumen diameter, mm			
In-stent	2.5 ± 0.46	2.46 ± 0.49 (1730)	0.19
In-segment	2.15 ± 0.51	2.06 ± 0.55 (1730)	0.006
Percent diameter stenosis, %			
In-stent	10.2 ± 7.5	10.7 ± 8.8 (1729)	0.26
In-segment	19.9 ± 10.8	22.5 ± 12.0 (1729)	0.002
Acute gain, mm			
In-stent	1.7 ± 0.53	1.65 ± 0.54 (1730)	0.1
In-segment	1.34 ± 0.56	1.24 ± 0.58 (1730)	0.002

Values are expressed as mean ± SD, median (interquartile range) or number (%)

SYNTAX score, synergy between percutaneous coronary intervention with taxus and cardiac surgery score

### Discontinuation of Thienopyridine

**Fig. 2:**
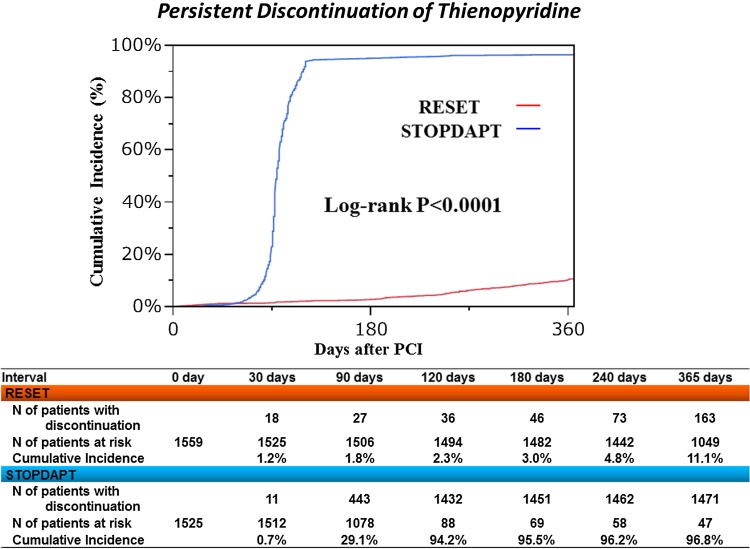
Cumulative incidence of persistent discontinuation of thienopyridine: STOPDAPT versus RESET

In the STOPDAPT, thienopyridine was discontinued within 4 months in 1444 patients (94.7 %). The reasons for not stopping thienopyridine within 4 months (protocol violation) in 81 patients included the decisions by the attending physician (16 patients), by the patient (8 patients), and by the general practitioner (33 patients), occurrence of events (14 patients; death: 4 patients, stroke: 3 patients, PCI: 6 patients, and peripheral artery disease: 1 patient), aspirin discontinuation (7 patients) and no hospital visit (3 patients). Cumulative 4-month and 1-year incidence of persistent discontinuation of thienopyridine was 94.2 and 96.8 %, respectively, in the STOPDAPT and 2.3 and 11.1 %, respectively, in the RESET (Fig. 2).

### Clinical outcomes through 1 year

**Fig. 3:**
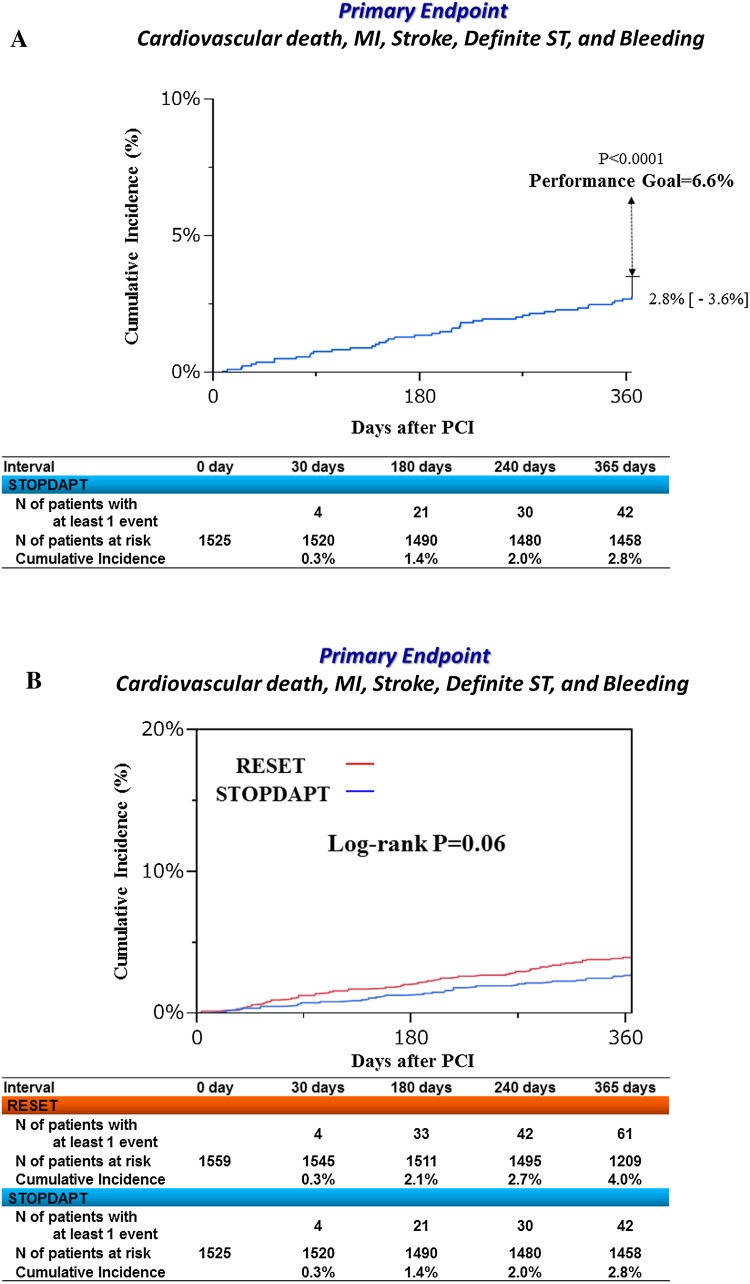
**a** Cumulative incidence of the primary endpoint. Primary endpoint, a composite of cardiovascular death, MI, stroke, definite ST and TIMI major/minor bleeding. **b** Cumulative incidence of the primary endpoint: STOPDAPT versus RESET. Primary endpoint, a composite of cardiovascular death, MI, stroke, definite ST and TIMI major/minor bleeding; *MI* myocardial infarction, *ST* stent thrombosis, *TIMI* thrombolysis in myocardial infarction

**Fig. 4:**
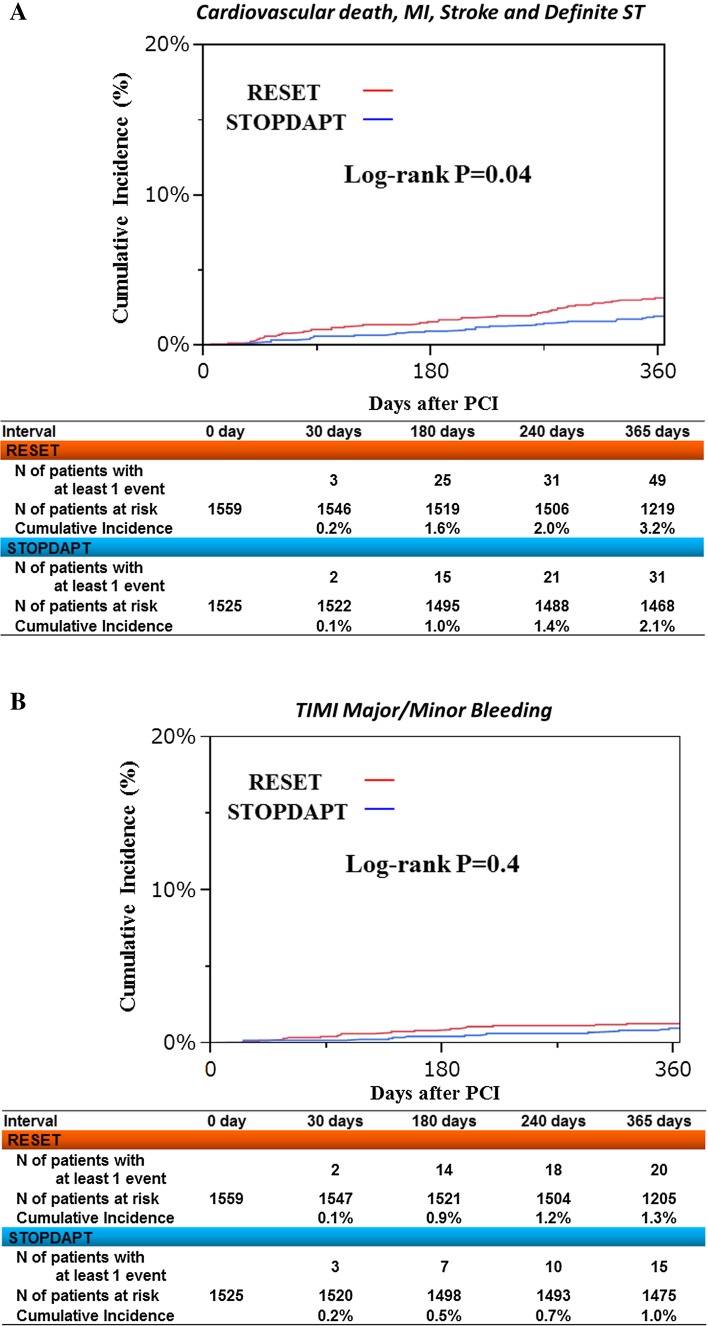
**a** Cumulative incidence of a composite of cardiovascular death, MI, stroke and definite ST: STOPDAPT versus RESET. *MI* myocardial infarction, *ST* stent thrombosis. **b** Cumulative incidence of TIMI major/minor bleeding: STOPDAPT versus RESET. *TIMI* thrombolysis in myocardial infarction

Complete 1-year clinical follow-up was achieved in 1519 patients (99.6 %) (Fig. 1). The cumulative 1-year incidence of the primary endpoint was 2.8 % (upper 97.5 % CI 3.6 %), which was significantly lower than the pre-defined performance goal of 6.6 % (*P* < 0.0001) (Fig. 3a). Cumulative 1-year incidence of the primary endpoint tended to be lower in the STOPDAPT than in the RESET (2.8 versus 4.0 %, *P* = 0.06) (Fig. 3b; Table 4). In the multivariable analysis, the risk for the primary endpoint was significantly lower in the STOPDAPT than in the RESET [adjusted HR 0.64 (95 % CI 0.42–0.95), *P* = 0.03] (S1 Table). The cumulative 1-year incidence of definite/probable ST was lower in the STOPDAPT than in the RESET [0 patient (0 %) versus 5 patients (0.3 %), *P* = 0.03] (Table 4). Regarding the major secondary endpoint, the cumulative incidence of a composite of cardiovascular death, MI, stroke and definite ST was significantly lower in the STOPDAPT than in the RESET, while the cumulative incidence of TIMI major/minor bleeding was not significantly different between the 2 groups (Fig. 4; Table 4).

**Table 4:** Clinical outcomes at 12 months

	No. of patients with at least one event (cumulative incidence)		*P* value
	STOPDAPT (*N* = 1525)	RESET (*N* = 1559)	
Primary Endpoint	42 (2.8 %)	61 (4.0 %)	0.06
Death			
All-cause	30 (2.0 %)	25 (1.6 %)	0.49
Cardiac death	9 (0.6 %)	13 (0.9 %)	0.4
Cardiovascular death	10 (0.7 %)	15 (1.0 %)	0.33
Non-cardiac death	21 (1.4 %)	12 (0.8 %)	0.11
Myocardial infarction	4 (0.3 %)	18 (1.2 %)	0.003
Stroke			
Any	17 (1.1 %)	21 (1.4 %)	0.51
Ischemic	14 (0.9 %)	15 (1.0 %)	0.86
Hemorrhagic	4 (0.3 %)	8 (0.5 %)	0.24
Bleeding			
TIMI major	12 (0.8 %)	12 (0.8 %)	0.99
TIMI minor/major	15 (1.0 %)	20 (1.3 %)	0.4
TIMI minimal/minor/major	37 (2.5 %)	38 (2.5 %)	0.9
GUSTO severe	10 (0.7 %)	16 (1.0 %)	0.23
GUSTO moderate/severe	16 (1.1 %)	19 (1.2 %)	0.61
Definite stent thrombosis			
All patients	0 (0 %)	4 (0.3 %)	0.046
Acute (0–1 day)	0 (0 %)	0 (0 %)	
Subacute (2–30 days)	0 (0 %)	1 (0.06 %)	
Late (31–365 days)	0 (0 %)	3 (0.2 %)	
Stent thrombosis			
Possible	6 (0.4 %)	7 (0.5 %)	0.78
Probable	0 (0 %)	1 (0.07 %)	0.32
Definite/probable	0 (0 %)	5 (0.3 %)	0.03
Definite/probable/possible	6 (0.4 %)	12 (0.8 %)	0.16
Death or myocardial infarction	34 (2.2 %)	40 (2.6 %)	0.49
Cardiovascular death or myocardial infarction	14 (0.9 %)	30 (2.0 %)	0.02
Cardiovascular death, MI or stroke	31 (2.1 %)	49 (3.2 %)	0.045
Cardiovascular death, MI, stroke and definite ST	31 (2.1 %)	49 (3.2 %)	0.045
Target-lesion revascularization	30 (2.0 %)	62 (4.2 %)	0.0007
Target-vessel revascularization	55 (3.7 %)	102 (6.9 %)	<0.0001
Coronary revascularization			
Any	109 (7.3 %)	175 (11.8 %)	<0.0001
Coronary artery bypass grafting	3 (0.2 %)	7 (0.5 %)	0.2

Values are expressed as number (%)

*TIMI* thrombolysis in myocardial infarction, *GUSTO* global utilization of streptokinase and tissue plasminogen activator for Occluded coronary arteries, *MI* myocardial infarction, *ST* stent thrombosis

**Fig. 5:**
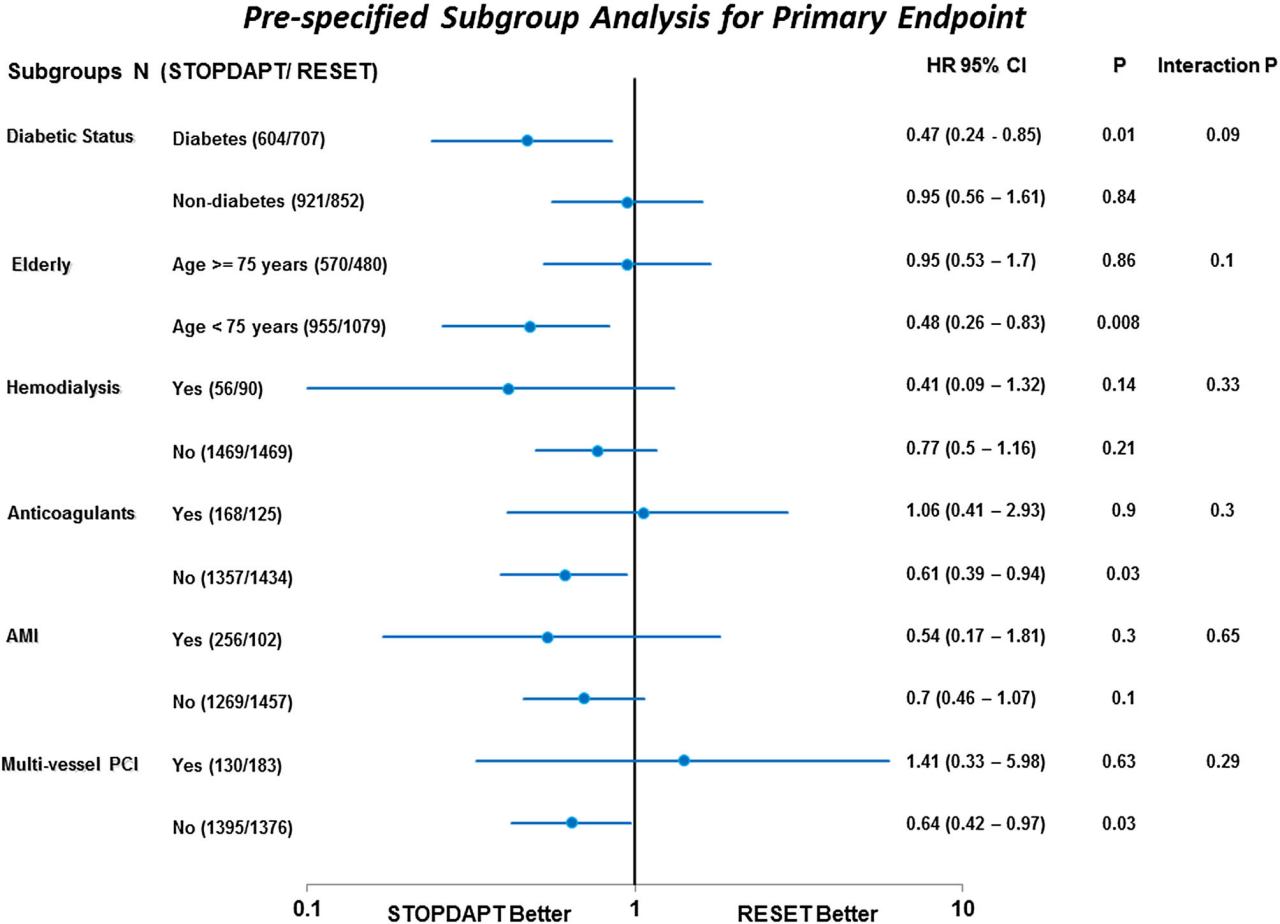
Forrest plot for the hazard ratios of STOPDAPT relative to RESET for the primary endpoint in the pre-specified subgroups. Primary endpoint, a composite of cardiovascular death, MI, stroke, definite ST and TIMI major/minor bleeding; *MI* myocardial infarction, *ST* stent thrombosis, *TIMI* thrombolysis in myocardial infarction, *AMI* acute myocardial infarction, *PCI* percutaneous coronary intervention

In the subgroup analysis, the STOPDAPT was associated with significantly lower risk for the primary endpoint compared with the RESET in those with diabetes and <75 years of age as well as those without anticoagulants and multivessel PCI. However, the interaction between the study (STOPDAPT or RESET) and the subgroup factor was not significant for any of the pre-specified subgroup factors (Fig. 5).

### Clinical outcomes between 3 and 12 months

Between 3 and 12 months, the cumulative incidence of the primary endpoint was not significantly different between the STOPDAPT and the RESET (2.0 versus 2.7 %, *P* = 0.19). No patients had definite or probable ST in the STOPDAPT, while 4 patients (0.3 %) had definite or probable ST in the RESET between 3 and 12 months (Table 5). The cumulative incidence of TIMI major/minor bleeding between 3 and 12 months was not significantly different between the 2 groups (Table 5).

**Table 5:** Clinical outcomes between 3 and 12 months

	No. of patients with at least one event (cumulative incidence)		*P* value
	STOPDAPT	RESET	
Primary Endpoint	30 (2.0 %)	41 (2.7 %)	0.19
Death			
All-cause	25 (1.7 %)	18 (1.2 %)	0.28
Cardiac death	9 (0.6 %)	13 (0.9 %)	0.4
Cardiovascular death	8 (0.5 %)	11 (0.7 %)	0.5
Non-cardiac death	17 (1.1 %)	8 (0.5 %)	0.07
Myocardial infarction	2 (0.1 %)	13 (0.9 %)	0.004
Stroke			
Any	11 (0.7 %)	11 (0.7 %)	0.97
Ischemic	9 (0.6 %)	8 (0.5 %)	0.82
Hemorrhagic	3 (0.2 %)	4 (0.3 %)	0.68
Bleeding			
TIMI major	10 (0.7 %)	7 (0.5 %)	0.48
TIMI minor/major	12 (0.8 %)	13 (0.9 %)	0.84
TIMI minimal/minor/major	26 (1.7 %)	25 (1.7 %)	0.92
GUSTO severe	7 (0.5 %)	10 (0.7 %)	0.44
GUSTO moderate/severe	11 (0.7 %)	12 (0.8 %)	0.81
Definite stent thrombosis	0 (0 %)	3 (0.2 %)	0.08
Stent thrombosis			
Possible	6 (0.4 %)	4 (0.3 %)	0.53
Probable	0 (0 %)	1 (0.07 %)	0.32
Definite/probable	0 (0 %)	4 (0.3 %)	0.045
Definite/probable/possible	6 (0.4 %)	8 (0.5 %)	0.59
Death or myocardial infarction	27 (1.8 %)	28 (1.9 %)	0.89
Cardiovascular death or myocardial infarction	10 (0.7 %)	21 (1.4 %)	0.049
Cardiovascular death, MI or stroke	21 (1.4 %)	32 (2.1 %)	0.13
Cardiovascular death, MI, stroke and definite ST	21 (1.4 %)	32 (2.1 %)	0.13
Target-lesion revascularization	29 (1.9 %)	57 (3.8 %)	0.002
Target-vessel revascularization	52 (3.5 %)	93 (6.3 %)	0.0004
Coronary revascularization			
Any	98 (6.6 %)	158 (10.8 %)	<0.0001
Coronary artery bypass grafting	3 (0.2 %)	6 (0.4 %)	0.31

Values are expressed as number (%)

Abbreviations are as in Table 4

## Discussion

The main finding of the current study is that stopping DAPT at 3 months in selected patients after CoCr-EES implantation was at least as safe as the prolonged DAPT regimen adopted in the historical control group.

Several previous randomized controlled trials compared 6-month versus ≥12-month DAPT after implantation of G1- and G2-DES, demonstrating similar ischemic event risk and lower bleeding event risk with 6-month DAPT [1, 2, 15–17]. Regarding the DAPT duration shorter than 6-month, 3-month DAPT with E-ZES (G1-DES) was non-inferior to 12-month DAPT with the other G1- or G2-DES with respect to the primary composite endpoints in the RESET and OPTIMIZE trials [3, 4]. In this first prospective study stopping DAPT at 3 months after CoCr-EES implantation, cumulative incidence of the primary endpoint was significantly lower than the pre-defined performance goal and tended to be lower than that in the historical control of the RESET, where nearly 90 % of patients continued DAPT at 1 year.

It was noteworthy that no definite or probable ST occurred in patients enrolled in the STOPDAPT. CoCr-EES is reported to be less thrombogenic compared with BMS by the bench testings [18]. In clinical trials and registries, the rates of late and very late ST were consistently very low after implantation of G2-DES, CoCr-EES in particular [7, 19, 20]. Given the extremely low incidence of late and very late ST, it might not be clinically appealing to extend DAPT duration to reduce the risk for ST. The cumulative 1-year incidences of cardiovascular death and MI were also very low with 3-month DAPT, which has also been demonstrated in the RESET and OPTIMIZE trials [3, 4]. Therefore, 3-month DAPT might be sufficient to protect patients from ischemic events within 1 year after implantation of G2-DES, if the patients have low ischemic event risk, like those enrolled in the current study.

The cumulative 1-year incidences of TIMI major/minor bleeding and other bleeding endpoints were not significantly different between the STOPDAPT and the RESET. Patients in the STOPDAPT included more patients with high bleeding risks such as advanced age, hypertension and anticoagulants usage than those in the RESET. The different bleeding risk profiles between the STOPDAPT and RESET trials might have led to the similar bleeding incidences between the 2 trials. In addition, the current study as well as the RESET and OPTIMIZE trials did not have enough statistical power to demonstrate the difference in the rates of bleeding events [3, 4]. However, shorter as compared with prolonged DAPT duration was clearly associated with lower risk of bleeding in the meta-analysis [5].

Recently, the DAPT trial demonstrated that 30-month DAPT, as compared with 12-month DAPT, reduced the rates of ST and major adverse cardiovascular and cerebrovascular events [21]. It might be important to distinguish the mandatory DAPT duration to protect patients against ST from long-term antiplatelet therapy as a secondary prevention. Considering the increased bleeding events and a signal suggesting increasing mortality [21], systematic implementation of prolonged DAPT would not be appropriate. The mandatory DAPT duration after coronary stent implantation would remain to be shorter than 1 year. We should continue to ask who would be the appropriate candidates for intensive long-term antiplatelet therapy, and what would be the optimal long-term antiplatelet regimen.

### Study limitation

There are several important limitations in the current study. First, and most importantly, this study was not a randomized controlled trial, but a single-arm study comparing with a historical control group. We could not draw any definitive conclusions from a single-arm study. The current study was designed as a pilot study to investigate the safety of 3-month DAPT in patients receiving G2-DES, because the study sponsor had planned a large randomized controlled trial comparing 3 months versus longer DAPT duration after G2-DES implantation. Second, selection bias toward inclusion of patients with lower ischemic risk should be considered when interpreting the result of this study. Multivariable analysis could not fully adjust the measured and unmeasured confounders. Third, detailed information of PCI such as final balloon size, balloon dilatation pressure and intravascular ultrasound use was not collected in this study. Finally, we could not exclude the possibility of underreporting of the clinical events in this investigator-driven study. However, the method of follow-up data collection was exactly the same in the STOPDAPT as in the RESET.

## Conclusion

Stopping DAPT at 3 months in selected patients after CoCr-EES implantation was at least as safe as the prolonged DAPT regimen adopted in the historical control group.

## Electronic supplementary material

Below is the link to the electronic supplementary material.

Supplementary material 1 (DOCX 30 kb)
